# Seizure As the Sentinel Event: An Atypical Presentation of Anti-N-Methyl-D-Aspartate (NMDA) Receptor Encephalitis in a Young Adult Male

**DOI:** 10.7759/cureus.111653

**Published:** 2026-06-28

**Authors:** Nicholas J Hendee, James C Mooney, Amy E Brzuchalski, Edward E Bridges

**Affiliations:** 1 Internal Medicine, Dwight D. Eisenhower Army Medical Center, Fort Gordon, USA; 2 Psychiatry, Dwight D. Eisenhower Army Medical Center, Fort Gordon, USA

**Keywords:** anti-nmda encephalitis, autoimmune encephalitis, neurology, new-onset seizure, nmda receptor, psychosis

## Abstract

This case report details an atypical presentation of anti-N-methyl-D-aspartate (NMDA) receptor encephalitis in a 21-year-old male, characterized by a "seizure-first" phenotype. The patient’s initial generalized tonic-clonic seizure led to a misdiagnosis of epilepsy, and subsequent psychiatric symptoms were incorrectly attributed to the side effects of antiepileptic medication or a new, undiagnosed psychiatric disorder. This resulted in an involuntary psychiatric admission and a delay in diagnosis and, thus, definitive treatment. Once the Graus criteria for probable anti-NMDA receptor encephalitis were met (presence of at least four out of six neuropsychiatric symptom categories, an abnormal electroencephalogram (EEG), and the reasonable exclusion of alternative causes), the patient was empirically treated with high-dose steroids. Upon detecting anti-NMDA receptor antibodies at a serum titer of 1:1000 and a cerebrospinal fluid (CSF) titer of 1:100, the treatment regimen was escalated to include plasmapheresis. In this patient's case, earlier consideration of autoimmune encephalitis during his initial presentation of new-onset seizures and rapid psychiatric decline could have prevented the diagnostic delay and involuntary psychiatric admission.

## Introduction

Anti-N-methyl-D-aspartate (NMDA) receptor encephalitis is a rare, autoimmune-mediated inflammatory disorder of the central nervous system. This neuropsychiatric condition has an estimated annual incidence of 1.5 cases per million individuals, exhibits a distinct four-to-one female-to-male predominance, and primarily affects children and young adults [1]. The NMDA receptor is predominantly located in the hippocampus, cerebral cortex, and basal ganglia [2]. Under normal physiologic conditions, the NMDA receptor is postsynaptic, acting as a coincidence detector requiring glutamate and either serine or glycine binding and concomitant postsynaptic depolarization [2]. When this occurs, calcium influx triggers the regulation of memory and cortical function [2]. The pathogenesis of anti-NMDA receptor encephalitis involves IgG antibodies targeting the GluN1 subunit of the NMDA receptor [3]. This results in internalization of the NMDA receptors and impaired inhibition of the dopaminergic and mesolimbic pathways, causing psychiatric and neurologic manifestations [3]. In patients with anti-NMDA receptor encephalitis, symptoms are generally reversible with treatment, but can progress to permanent damage due to NMDA-mediated glutamate excitotoxicity with lasting neuropsychiatric symptoms if treatment is delayed [4]. Anti-NMDA receptor encephalitis is frequently associated with an underlying malignancy, most commonly an ovarian teratoma, which is identified in approximately 40% to 50% of female patients aged 18 to 45 [1,5]. The Graus criteria can be used to clinically identify cases of probable anti-NMDA receptor encephalitis by satisfying the following criteria: rapid onset of neurologic or psychiatric symptoms in four out of six categories (speech dysfunction, seizures, abnormal behavior, movement disorders including rigidity or posturing, autonomic dysfunction), abnormal electroencephalogram (EEG), or abnormal cerebrospinal fluid (CSF) with oligoclonal banding or pleocytosis, and reasonable exclusion of other disorders [6].

In young adults, typical symptoms progress in stages, the first being a viral-like prodrome of nonspecific fever, aches, chills, and malaise, followed by psychiatric manifestations, including hallucinations, agitation, and acute psychosis [1,4,7]. These psychiatric symptoms often precede neurologic symptoms, such as altered mentation, focal deficits, and seizures, and can culminate in unresponsiveness [1,4,7]. Approximately 80% of cases of anti-NMDA receptor encephalitis in young adults will present in this sequence [8]. Conversely, in children and adolescents, anti-NMDA receptor encephalitis more commonly presents with neurological symptoms preceding psychiatric [7,9]. Although the literature documents cases of adult male patients presenting initially with seizures [10], these are more commonly focal (partial) seizures [11] rather than the generalized tonic-clonic seizure observed in our patient.

This case highlights an atypical presentation of anti-NMDA receptor encephalitis in a young adult, where an initial seizure preceded the subsequent development of psychiatric symptoms, including agitation, hallucinations, and acute psychosis. Because of this unusual onset, the patient was initially misdiagnosed, left the hospital against medical advice, and underwent an involuntary psychiatric admission, ultimately delaying correct diagnosis and appropriate treatment.

## Case presentation

A 21-year-old male presented to the emergency department following a witnessed, first-time seizure. His roommate observed a two-minute episode characterized by upper-body myoclonus and loss of consciousness. The patient arrived at the emergency department immediately following the episode. He had no previous medical or psychiatric history and denied any recent viral-like illness or febrile episodes. On presentation, the patient was hemodynamically stable, fully alert, and oriented. His neurological exam revealed intact cranial nerves and symmetric motor strength in all extremities. Laboratory evaluation was unrevealing for metabolic or toxic etiologies of seizure (Table 1).

**Table 1:** Venous blood gas, complete blood count, comprehensive metabolic panel, and toxicology screening (ammonia, ethanol, salicylate, acetaminophen, and urine) obtained at initial emergency department presentation POC: point of care

Laboratory Test	Result	Reference Range	Interpretation
POC pH (vein)	7.409	7.31 - 7.41	-
POC pCO2 (vein)	36 mmHg	40 - 50 mmHg	-
POC pO2 (vein)	38 mmHg	30 - 50 mmHg	-
POC HCO3 (vein)	23 mEq/L	22 - 29 mEq/L	-
POC Hgb (vein)	16.3 g/dL	13.8 - 17.2 g/dL	-
WBC	8.00 10*3/μL	4.5 - 11.0 10*3/μL	-
RBC	5.04 106*/μL	4.5 - 5.9 10*6/μL	-
Hemoglobin	14.5 g/dL	13.8 - 17.2 g/dL	-
Hematocrit	43.4%	41% - 50%	-
MCV	86.2 fL	80 - 100 fL	-
Platelets	277 103/μL	150 - 450 103/μL	-
Sodium	140.0 mmol/L	135 - 145 mmol/L	-
Potassium	3.7 mmol/L	3.5 - 5.2 mmol/L	-
Chloride	104 mmol/L	96 - 106 mmol/L	-
CO2	24 mmol/L	23 - 29 mmol/L	-
Anion Gap	12 mmol/L	8-16 mmol/L	-
BUN	10 mmol/L	7 - 20 mg/dL	-
Creatinine	1.0 mmol/L	0.7 - 1.3 mg/dL	-
eGFR	110 mL/min/1.73 m2	> 90 mL/min/1.73 m2	-
Glucose	87 mg/dL	70 - 100 mg/dL	-
Calcium	9.7 mg/dL	8.6 - 10.3 mg/dL	-
Protein (total)	7.9 g/dL	6.0 - 8.3 g/dL	-
Albumin	4.8 g/dL	3.5 - 5.0 g/dL	-
Phosphorus	4.3 mg/dL	2.8 - 4.5 mg/dL	-
Magnesium	2.1 mg/dL	1.7 - 2.2 mg/dL	-
Alk Phos	68 U/L	44 - 147 U/L	-
ALT	32 U/L	7 - 56 U/L	-
AST	26 U/L	10 - 40 U/L	-
Lactic Acid	1.2 mmol/L	0.8-2.8 mmol/L	-
Ammonia Lvl	18 mmol/L	10.0 - 47.0 mmol/L	-
Ethanol Lvl	<10 mg/dL	0.0 - 10.0 mg/dL	-
Salicylate Lvl	<5 mg/dL	1.0 - 10.0 mg/dL	-
Acetaminophen Lvl	<3.0 mcg/ml	10.0 - 30.0 mcg/ml	Low
Urine Amphetamine	Negative	Negative	-
Urine Barbiturate	Negative	Negative	-
Urine Benzodiazepine	Negative	Negative	-
Urine Cannabinoids	Negative	Negative	-
Urine Cocaine	Negative	Negative	-
Urine Methadone	Negative	Negative	-
Urine Opiate	Negative	Negative	-
Urine Oxycodone	Negative	Negative	-
Urine Phencyclidine	Negative	Negative	-
Urine Fentanyl	Negative	Negative	-

A computed tomography (CT) scan of the head without contrast demonstrated a posterior fossa area of CSF attenuation measuring 8 cm x 3 cm consistent with an arachnoid cyst without mass effect or midline shift, determined to be incidental and non-contributory (Figures 1A-1C). Based on the roommate’s description, the patient was believed to have suffered a generalized tonic-clonic seizure. He received a single dose of intravenous (IV) levetiracetam 2,000 mg and was prescribed oral levetiracetam 1,000 mg twice daily and was discharged with outpatient neurology follow-up. Over the following week, the patient returned to the emergency department twice for near-syncopal episodes without further seizure activity. After an unremarkable physical exam and laboratory evaluation, he was discharged both times. A timeline detailing the patient's multiple ED presentations and inter-facility transfers is included (Figure 2).

**Figure 1:**
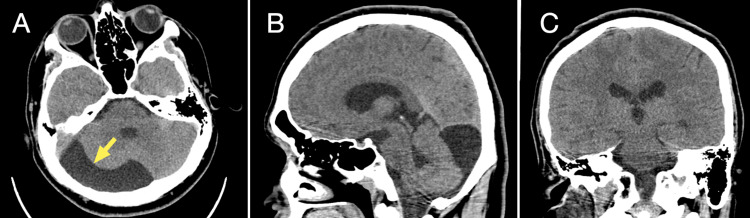
CT scan of the head obtained during the patient’s initial emergency department presentation (Panel A) Axial, (Panel B) sagittal, and (Panel C) coronal views demonstrate an 8 cm x 3 cm area of cerebrospinal fluid (CSF) attenuation in the right posterior fossa (yellow arrow), consistent with an arachnoid cyst. There is no associated mass effect or midline shift. These findings were considered incidental and non-contributory to the patient's acute presentation.

**Figure 2:**
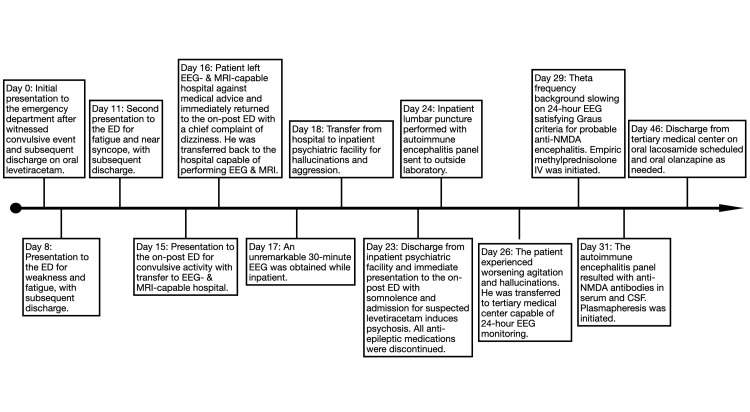
A timeline illustrating the patient's multiple emergency department presentations, subsequent inter-facility transfers, and initiation of empiric therapy for anti-NMDA encephalitis. NMDA: N-methyl-D-aspartate

Fifteen days after the initial presentation, the patient returned following a second convulsive episode. A witness described it as "shaking all over" for approximately one minute, followed by a period of unconsciousness. On presentation, he was hemodynamically stable, somnolent but arousable, alert, oriented, and without cranial nerve deficits. He was transferred to an external hospital facility for magnetic resonance imaging (MRI) and 24-hour EEG; however, due to escalating paranoia and agitation, he prematurely left against medical advice (AMA). He returned to the emergency department less than 24 hours later with complaints of dizziness and profound fatigue, at which point he was readmitted to complete the diagnostic workup.

During this admission, the patient developed near-continuous intense auditory and visual hallucinations with an autonomic response of tachycardia and hypertension, agitation, and aggressiveness, ultimately requiring restraints. MRI redemonstrated the incidental arachnoid cyst, and a 30-minute EEG was completed, which demonstrated beta waves and an absence of any epileptiform activity. Given an unremarkable MRI and EEG thus far alongside worsening psychiatric symptoms, the patient was involuntarily transferred to an inpatient psychiatric facility 18 days after his initial presentation.

At the psychiatric facility, the patient suffered two more convulsive episodes and developed progressive somnolence, tangential thoughts, and profound hallucinations. His medication regimen was heavily altered. Levetiracetam was replaced briefly by oral divalproex sodium 125 mg twice daily, and he received as-needed lorazepam and olanzapine for agitation. By discharge, all antiepileptics and sedatives were discontinued in favor of oral olanzapine 5 mg twice daily.

Six days later (23 days after the initial presentation), the patient was discharged to his parents, who brought him to the emergency department for his sixth presentation. They reported that he was now excessively drowsy and experiencing persecutory hallucinations. On examination, the patient was hemodynamically stable, alert to verbal stimuli, and oriented only to person and place. He reported visual and auditory hallucinations and exhibited episodic autonomic instability manifesting as tachycardia and tachypnea. His mental status waxed and waned, alternating between stupor, disorganization, non-aggressive emotional lability, and confusion. Laboratory evaluation was unremarkable except for a urine drug screen positive for benzodiazepines, which had been administered at the psychiatric facility. Psychiatry consultation recommended medical admission with a neurology consult for further evaluation of the medical etiologies of what phenotypically presented as delirium. The primary differential diagnosis encompassed over-sedation secondary to polypharmacy, levetiracetam-induced psychotic disorder, delirium, and acute intracranial insult, with possible autoimmune encephalitis representing a leading clinical concern. A primary psychiatric disorder and nonconvulsive status epilepticus (NCSE) were considered unlikely components of the initial differential diagnosis. To systematically distinguish an iatrogenic encephalopathy from an active neuroinflammatory process, a medication washout period was initiated upon admission, during which both levetiracetam and olanzapine were discontinued. Given the low clinical index of suspicion for NCSE relative to a presumed medication-induced effect, transfer to a tertiary facility was deferred.

During the early morning hours of day 24, the patient experienced insomnia, episodic agitation, hallucinations with associated autonomic instability consisting of tachycardia up to 182 beats per minute, hypertension with pressures to 193/104 mmHg, and tachypnea without further seizure activity. Lumbar puncture was performed successfully the following morning, and an autoimmune encephalitis panel was obtained. Basic CSF analysis revealed a normal opening pressure alongside an elevated red blood cell count and lymphocytic predominance without pleocytosis. Protein and glucose concentrations were within normal ranges, molecular polymerase chain reaction (PCR) testing for infectious etiologies was negative, and oligoclonal bands were absent (Table 2). The patient continued to decline with increasing intensity of paranoia, hallucinations, and aggression, requiring continued redirection and one-to-one monitoring.

**Table 2:** Initial lumbar puncture cerebrospinal fluid results CSF: cerebrospinal fluid; PCR: polymerase chain reaction

Laboratory Test	Result	Reference Range	Interpretation
CSF Color	Colorless	Colorless	-
CSF Clarity	Clear	Clear	-
CSF WBC	2 cells/mcL	0.0 - 8.0 cells/mcL	-
CSF RBC	14 cells/mcL	0 cells/mcL	High
CSF cells counted	100	-	-
CSF segs	3%	0 - 6%	-
CSF lymphocytes	95%	40 - 80%	High
CSF monocyte	1%	15 - 45%	Low
CSF eosinophils	1%	<1%	-
CSF glucose	61 mg/dL	40.0 - 80.0 mg/dL	-
CSF protein	26.2 mg/dL	15.0 - 45.0 mg/dl	-
Opening Pressure	15 cm H2O	10-20 cm H2O	-
Oligoclonal Bands	0	<4	-
Meningitis PCR Panel	Not detected	Not detected	-
Cryptococcus PCR	Not detected	Not detected	-
Varicella PCR	Not detected	Not detected	-
Herpes Simplex 1 PCR	Not detected	Not detected	-
Herpes Simplex 2 PCR	Not detected	Not detected	-
Herpes Simplex 6 PCR	Not detected	Not detected	-
Cytomegalovirus PCR	Not detected	Not detected	-
Neisseria meningitidis PCR	Not detected	Not detected	-
Listeria monocytogenes PCR	Not detected	Not detected	-
Strep pneumoniae PCR	Not detected	Not detected	-

Following the medication washout period, the persistence and escalation of the patient's profound psychiatric and autonomic symptoms effectively excluded over-sedation and levetiracetam-induced psychosis from the differential diagnosis. This clinical decline in the absence of pharmacologic triggers, paired with the unrevealing infectious workup and basic CSF analysis, shifted the diagnostic focus away from a primary delirium or medication adverse effect. Instead, the constellation of progressive encephalopathy, intractable hallucinations, and autonomic instability strongly pointed toward a rapidly evolving primary neurologic process, making autoimmune encephalitis or non-convulsive status epilepticus the leading concerns.

Due to a continued decline in mental status, completion of the medication washout, and evolving differential diagnosis, the patient was transferred to a tertiary hospital for 24-hour EEG monitoring on day 26. A repeat MRI was performed, but it was terminated early due to patient agitation. The EEG was significant for frequent generalized periodic discharges with triphasic morphology at 1 to 1.5 Hz and generalized theta-frequency background slowing consistent with non-specific diffuse or multifocal cerebral dysfunction. One electrographic seizure was observed, characterized by high-amplitude, sharp theta evolving to delta frequencies without clear laterality. During this event, the patient experienced a period of decreased responsiveness to family, myoclonus of the jaw, and subsequent tonic progressing to clonic movements of all extremities. This episode lasted less than five minutes and stopped abruptly.

Revisiting the Graus criteria for autoimmune encephalitis, the patient met five of the six neuropsychiatric symptom categories within three months, presenting with a rapid onset of psychiatric behavioral changes, pressured and slurred speech, seizures, memory impairment, and autonomic dysfunction. Combined with the now abnormal EEG, empiric treatment for probable autoimmune encephalitis was warranted, and 29 days after initial presentation, the patient was started on IV methylprednisolone 1,000 mg daily for 7 days. Given the known association between autoimmune encephalitis and malignancy, the patient underwent an extensive paraneoplastic workup consisting of a CT scan of the chest, abdomen, and pelvis and an ultrasound of the scrotum.

Ultimately, the autoimmune encephalitis panel confirmed the presence of anti-NMDA receptor antibodies with a serum titer of 1:1,000 and a CSF titer of 1:100 (Table 3 and Table 4). Given these new results and continued altered mental status requiring intermittent physical restraints, 5 cycles of every other day plasmapheresis were initiated 31 days after initial presentation. Extensive paraneoplastic screening was negative; CT imaging of the chest, abdomen, and pelvis (Figures 3, 4) and a scrotal ultrasound (Figure 5) revealed no evidence of malignancy or discrete masses. The patient demonstrated moderate symptomatic improvement after a course of methylprednisolone and plasmapheresis. He was persistently somnolent but arousable with occasional paranoia, but without hallucinations. Slurred speech was present, although improved, and he no longer had episodes of autonomic instability. IV methylprednisolone and plasmapheresis courses were completed and discontinued 36 and 41 days after presentation, respectively, without further witnessed seizure episodes. He was transitioned to oral lacosamide 100 mg twice daily for seizure prophylaxis and oral olanzapine 5 mg as needed for agitation, and was finally discharged 46 days after initial presentation. Ongoing outpatient care and follow-up were with further plans in place for outpatient intravenous immunoglobulin (IVIG) administration.

**Table 3:** Cerebrospinal fluid autoimmune encephalitis panel results Ab: antibody; IFA: indirect fluorescent antibody

Test Description	Result	Interpretation	Reference Range
Anti-Hu Ab	Negative	-	Negative
Anti-Ri Ab	Negative	-	Negative
Antineuronal nuclear Ab Type 3	Negative	-	Negative
PCA Type-1 (Anti-Yo) Ab	Negative	-	Negative
Purkinje Cell Cyto Ab Type 2	Negative	-	Negative
Purkinje Cell Cyto Ab Type Tr	Negative	-	Negative
Amphiphysin Antibody	Negative	-	Negative
CRMP-5 IgG	Negative	-	Negative
AGNA-1	Negative	-	Negative
DPPX Antibody	Negative	-	Negative
mGluR1 Antibody	Negative	-	Negative
IgLON5 Antibody	Negative	-	Negative
Ma2/Ta Antibody	Negative	-	Negative
Zic4 Antibody	Negative	-	Negative
DNER Antibody	Negative	-	Negative
ITPR1 Antibody	Negative	-	Negative
AMPA-R-1 Cell-based IFA	Negative	-	Negative
AMPA-R-2 Cell-based IFA	Negative	-	Negative
GABA-B Receptor Cell-based IFA	Negative	-	Negative
NMDAR Antibody, Cell-based IFA	Positive	Abnormal	Negative
Anti-NMDAR Antibody Titer	1:100	-	Neg: <1:10
GAD65 Antibody, Cell-based IFA	Negative	-	Negative
CASPR2 Antibody, Cell-based IFA	Negative	-	Negative
LGI1 Antibody, Cell-based IFA	Negative	-	Negative

**Table 4:** Serum autoimmune encephalitis panel results Ab: antibody; IFA: indirect fluorescent antibody

Test Description	Result	Interpretation	Reference Range
Anti-Hu Ab	Negative	-	Negative
Anti-Ri Ab	Negative	-	Negative
Antineuronal nuclear Ab Type 3	Negative	-	Negative
PCA Type-1 (Anti-Yo) Ab	Negative	-	Negative
Purkinje Cell Cyto Ab Type 2	Negative	-	Negative
Purkinje Cell Cyto Ab Type Tr	Negative	-	Negative
Amphiphysin Antibody	Negative	-	Negative
CRMP-5 IgG	Negative	-	Negative
AGNA-1	Negative	-	Negative
DPPX Antibody	Negative	-	Negative
mGluR1 Antibody	Negative	-	Negative
IgLON5 Antibody	Negative	-	Negative
Ma2/Ta Antibody	Negative	-	Negative
Zic4 Antibody	Negative	-	Negative
DNER Antibody	Negative	-	Negative
ITPR1 Antibody	Negative	-	Negative
AMPA-R1 Antibody	Negative	-	Negative
AMPA-R2 Antibody	Negative	-	Negative
GABA-B-R Antibody	Negative	-	Negative
NMDA-R Antibody	Positive	Abnormal	Negative
NMDAR Antibody, Cell-based IFA	1:1000	-	Negative
GAD65 Antibody	Negative	-	Negative
CASPR2 Antibody, Cell-based IFA	Negative	-	Negative
LGI1 Antibody, Cell-based IFA	Negative	-	Negative

**Figure 3:**
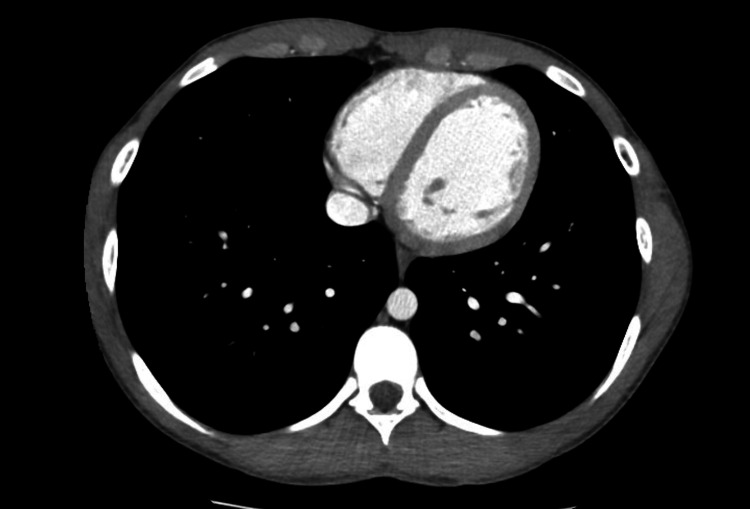
CT scan of the chest showing no evidence of mass or malignancy

**Figure 4:**
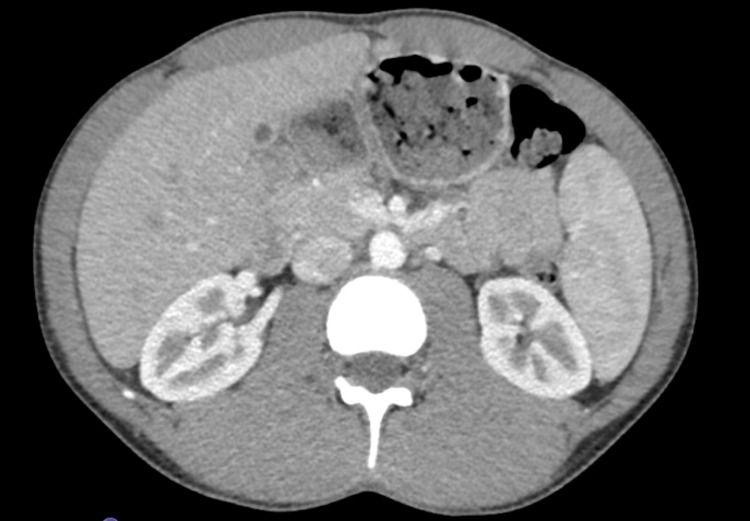
CT scan of the abdomen and pelvis showing a mild-to-moderate stool burden, with no evidence of mass or malignancy

**Figure 5:**
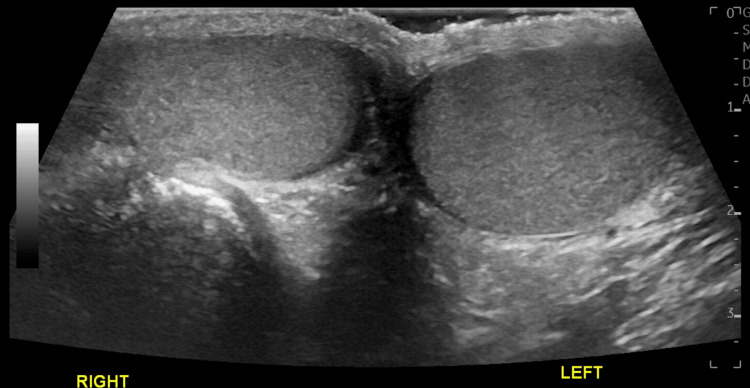
Ultrasound of the scrotum showing no evidence of mass or malignancy

At his 1-week post-discharge follow-up (53 days after initial symptom onset), the patient continued to improve and exhibited mild cognitive deficits, including difficulties with memory and information processing, and required assistance with activities of daily living (ADLs). Notably, he experienced dense retrograde amnesia, with no recollection of his subsequent hospitalization. According to his parents, the patient remained compliant with lacosamide, with no recurrent seizures, and did not require olanzapine for agitation. Outpatient management also included a single 80-gram dose of IVIG administered 17 days post-discharge (63 days after symptom onset), which he tolerated without adverse reaction.

Three months after discharge, the patient has had substantial neurological and psychiatric recovery with methylprednisolone, plasmapheresis, and IVIG. He is currently taking oral lacosamide and no longer requires olanzapine for agitation. He continues to have a mildly impaired thought process and memory with mild slurred speech; however, these are continuing to improve. He still requires some assistance completing ADLs, and the patient’s parents report no further convulsive activity or hallucinations. Episodes of agitation are much milder and redirectable, and they note a marked improvement in his circadian rhythm.

## Discussion

This case underscores the diagnostic challenges of anti-NMDA receptor encephalitis when it lacks the classic psychiatric prodrome observed in 80% of adult presentations [1]. The patient initially presented with a generalized tonic-clonic seizure, was misdiagnosed with epilepsy, and was prescribed oral levetiracetam. The subsequent psychiatric decline was further incorrectly attributed to medication side effects. This atypical, seizure-first presentation masked the underlying neuroinflammatory process, initiating a cascade of clinical misattributions that ultimately led to an inappropriate involuntary psychiatric admission and a critical diagnostic delay.

At the patient’s final hospital admission, the differential diagnosis favored levetiracetam-induced psychosis, over-sedation, or delirium; however, autoimmune encephalitis remained a strong consideration, while non-convulsive status epilepticus and a primary psychiatric disorder were less likely. The patient satisfied the neuropsychiatric symptom categories of the Graus criteria for probable autoimmune encephalitis; however, a previously obtained MRI and 30-minute EEG were unremarkable, and a comprehensive exclusion of alternative causes was incomplete. To systematically differentiate between an iatrogenic etiology and an active neuroinflammatory process, an intentional medication washout period was initiated. Had the symptoms been iatrogenic, withdrawal of levetiracetam should have yielded clinical improvement. Instead, when symptoms continued to worsen, an iatrogenic etiology was essentially eliminated from the differential. Focus then shifted to a primary neurological process, prompting a lumbar puncture and continuous 24-hour EEG monitoring to investigate for autoimmune encephalitis or nonconvulsive status epilepticus.

A teaching point in this case is the role and limitations of EEG in autoimmune encephalitis. Literature demonstrates that tracings can be normal in up to 10% of cases, making it unreliable to rule out suspected disease [12]. Furthermore, 91% of abnormal EEGs demonstrate only non-specific diffuse slowing rather than definitive epileptiform activity [12]. Similar findings were observed in our patient. His initial, 30-minute EEG was unremarkable, only capturing a brief snapshot of the entire clinical picture, and directly contributed to the involuntary psychiatric admission and diagnostic delay. To rule out nonconvulsive status epilepticus, a 24-hour EEG was later performed, revealing generalized theta-frequency background slowing and one captured seizure. This crucial finding allowed the patient to fulfill the Graus criteria for probable autoimmune encephalitis and prompted empiric treatment with IV corticosteroids. While the abnormal EEG supported an active neuroinflammatory process, the definitive diagnosis of anti-NMDA receptor encephalitis was confirmed only after correlating the evolving clinical picture with a positive cerebrospinal fluid autoantibody panel.

## Conclusions

While anti-NMDA receptor encephalitis is increasingly recognized in neurology, its tendency to mimic primary psychiatric disorders or idiopathic epilepsy continues to contribute to significant delays in definitive treatment. This risk is emphasized in male patients, where the clinical suspicion of autoimmune etiology may be lower given the absence of ovarian teratomas. This case highlights the "seizure-first" phenotype of anti-NMDA receptor encephalitis, a significant deviation from the classic psychiatric prodrome expected in young adults. The atypical sequence of symptoms led to a 29-day delay in initiating immunomodulatory therapy. Clinicians must maintain a high index of suspicion for autoimmune encephalitis in young adults presenting with new-onset seizures followed by rapid psychiatric decline, as early intervention remains the primary determinant of long-term functional and cognitive outcomes.
